# Population Structure of Mountain Pine Beetle Symbiont *Leptographium longiclavatum* and the Implication on the Multipartite Beetle-Fungi Relationships

**DOI:** 10.1371/journal.pone.0105455

**Published:** 2014-08-25

**Authors:** Clement Kin-Ming Tsui, Lina Farfan, Amanda D. Roe, Adrianne V. Rice, Janice E. K. Cooke, Yousry A. El-Kassaby, Richard C. Hamelin

**Affiliations:** 1 Department of Forest and Conservation Sciences, University of British Columbia, Vancouver, British Columbia, Canada; 2 Department of Biological Sciences, University of Alberta, Edmonton, Alberta, Canada; 3 Natural Resources Canada, Canadian Forest Services, Laurentian Forestry Centre, Québec City, Québec, Canada; USDA ARS, United States of America

## Abstract

Over 18 million ha of forests have been destroyed in the past decade in Canada by the mountain pine beetle (MPB) and its fungal symbionts. Understanding their population dynamics is critical to improving modeling of beetle epidemics and providing potential clues to predict population expansion. *Leptographium longiclavatum* and *Grosmannia clavigera* are fungal symbionts of MPB that aid the beetle to colonize and kill their pine hosts. We investigated the genetic structure and demographic expansion of *L. longiclavatum* in populations established within the historic distribution range and in the newly colonized regions. We identified three genetic clusters/populations that coincide with independent geographic locations. The genetic profiles of the recently established populations in northern British Columbia (BC) and Alberta suggest that they originated from central and southern BC. Approximate Bayesian Computation supports the scenario that this recent expansion represents an admixture of individuals originating from BC and the Rocky Mountains. Highly significant correlations were found among genetic distance matrices of *L. longiclavatum*, *G. clavigera*, and MPB. This highlights the concordance of demographic processes in these interacting organisms sharing a highly specialized niche and supports the hypothesis of long-term multipartite beetle-fungus co-evolutionary history and mutualistic relationships.

## Introduction

Mutualistic symbioses are strong evolutionary drivers, enabling specialization and diversification of host and microbial symbionts [1], [2], [3], [4]. Many insects are able to exploit and occupy ecological niches, inaccessible to other organisms, through symbiotic associations with micro-organisms [5], [6]. Molecular ecology approaches can help us to better understand these associations. In particular, studying whether the host shapes the microbial symbiont population structure would help our understanding of the dynamics of symbiotic associations. Observation of genetic concordance between symbionts and their hosts' life history features confirmed the hypothesis that species-specific, vertically transmitted symbionts exhibit population genetic structures consistent with that of their host [7], [8], [9]. Other studies further suggested that the population genetic structure and spatial distribution of symbionts may be linked to host/vector movement in the landscape [10]. This information is important for elucidating co-evolutionary relationships between hosts and microbial symbionts. If they are pathogens or infectious diseases, the approach of testing concordance through their genetic structure can increase our understanding of how pathogens/diseases are transmitted or spread over large spatial scales.

The bark beetle-microbe system represents a complex community of symbionts, which provides an opportunity to study the impact of multipartite symbiont associations on population structure, ecosystem health, and pest management [11], [12], [13]. Representatives of *Dendroctonus* are serious forest pests in North America. The mountain pine beetle (MPB; *Dendroctonus ponderosae* Hopkins) forms complex multipartite symbioses with many microbes including bacteria, single cell yeasts, and filamentous fungi (both Basidiomycota and Ascomycota) that include many tree pathogens [12], [14]. This species is of particular importance because of its large-scale population expansion and the devastation to its primary host lodgepole pine (*Pinus contorta* Douglas ex Loudon) and other conifer species [15]. The epidemic has had serious, region-wide economic implications due to substantial losses in wood volume. The destruction of large tracts of pine populations has led to increased emissions of CO_2_, which could generate a feedback loop of global warming which results in climatic conditions more conducive to further MPB population expansion [16].

MPB is a native pest in North America and it attacks many pine species in western North America [17]. The current outbreak started in the late 1990s, expanding extensively in southern British Columbia (BC), Canada, and spreading northward into northern BC and eastward into Alberta (AB). This epidemic is causing unprecedented devastation: more than 18 million ha of lodgepole pine forests in Canada have been affected by this outbreak (http://www.for.gov.bc.ca.hfp/mountain_pine_beetle/facts.htm) [16], [18]. Recently, the MPB has been shown to reproduce in pure jack pine (*P. banksiana* Lamb.) and has spread further east, attacking lodgepole × jack pine hybrids [19]. The extent and intensity of this MPB epidemic highlights the need to better understand the interactions and population dynamics between the MPB and its microbial symbionts [17].

When the MPB attacks pine trees, female adults bore through the bark and deposit microbes [15]; among these microbes are fungal symbionts (belonging to Ophiostomatales, Ascomycota) that can penetrate the sapwood and produce a blue stain discoloration in recently killed trees [12]. The presence of these fungi may affect wood structure and results in wood downgrading and loss of value. However, these fungi play important roles in the beetle life cycle by providing nutrition to the beetle's larvae, exhausting tree defenses, and modifying environmental conditions within the host trees [20], [21], [22]. Fungal associates/symbionts could double the concentration of nitrogen near the point of infection in the phloem of the pines. Mature beetles developing with these symbiotic fungi were greater in number in bolts from fertilized pines (nutrient supplemented) than those in unfertilized pines [23].

*Grosmmania clavigera* (Rob.-Jeffr. & R.W. Davidson) Zipfel, Z.W. de Beer & M.J. Wingf., *Leptographium longiclavatum* S.W. Lee, J.J. Kim & C. Breuil and *Ophiostoma montium* (Rumbold) Arx are the major ophiostomatoid fungi that appear to be exclusively associated with the MPB. Although these fungi share the same beetle host and colonize the same host trees, they have different pathogenic/virulence profiles and possibly roles. *G. clavigera* has been identified as a primary and aggressive invader of sapwood after beetle infestation and it is commonly isolated from the MPB mycangia, a special beetle adaptation for spore transport [24], [25], [26], [27], [28]. *Grosmannia clavigera* can kill mature or young lodgepole pine in the absence of the MPB when inoculated at a density similar to that of a beetle mass attack [28]. *Leptographium longiclavatum* also can cause necrotic tissue around inoculations point both on the phloem and the sapwood [29], while *O. montium* has been considered to be a weak pathogen [28], [30]. Apart from pathogenicity, these fungi also vary in genetic variability, relative abundance, spatial distribution pattern, and physiological characteristics [8], [31], [32]. Since fungal symbionts can affect beetle performance [11], [33], understanding their biology, ecology and population structure could help explain how the MPB became such a successful insect with the capacity to attack various pines over a broad geographic and climatic range.

The role of *L. longiclavatum* in the MPB-fungal interactions has received little attention; it has only been recently described as having previously been misidentified due to its similarity to *G. clavigera* [34], [35], [36]. Although *L. longiclavatum* and *G. clavigera* are closely related [35] and share similar morphological characters and physiological requirements [34], [36], *L. longiclavatum* has been relatively more abundant in the recently introduced MPB outbreak locations (northern BC and AB) when compared with the Rocky Mountains locations [31]. It is possible that *L. longiclavatum* is more adaptable/competitive in the cold boreal climate [37], leading to a gradual replacement of *G. clavigera* by *L. longiclavatum* with increasing latitude. It could be that niche competition is regulated by cold tolerance in these similar species [31]. Alternatively, it is possible that these fungi have partitioned the niche in a way that is not yet obvious.

**Figure 1:**
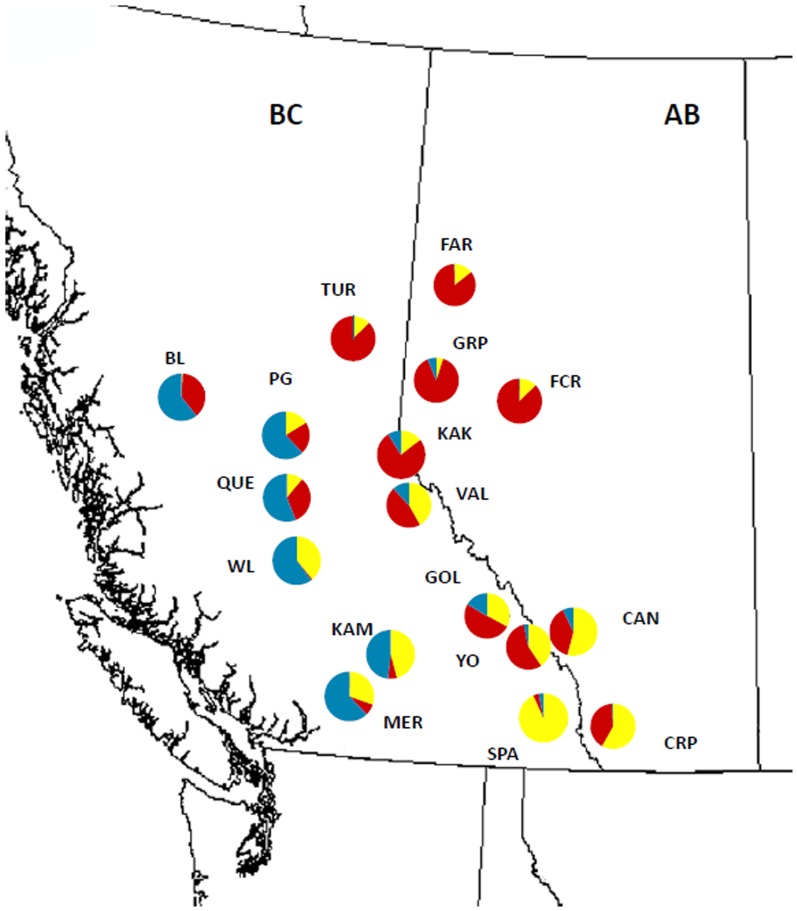
Population structure of *Leptographium longiclavatum* based on ten microsatellites using STRUCTURE. Each pie chart represents the average % assignment of fungal isolates (based on membership coefficient) from individuals into each of the three optimal clusters (“BC” by blue, “North” by red, “Rocky” by yellow) in each of the 17 populations.

*Grosmannia clavigera* populations formed four major genetic clusters (NBC: northern BC; SBC: southern BC; Rocky: the Rocky Mountains; North: epidemic populations in northern BC and AB) that resembled the observed north-south population differentiation pattern of MPB (Figure 1) in [38]. We hypothesize that the ecological relationship between *L. longiclavatum* and MPB is similar to that of *G. clavigera* and MPB, given the evolutionary, morphological, and physiological similarities between *L*. *longiclavatum* and *G. clavigera* [29], [32], [37]. Understanding the interactions between *L. longiclavatum* and MPB population structure may help elucidate some aspects of the MPB outbreak, including population size, founder effect, and migration patterns, which is information that could help us to model outbreak dynamics. Also the frequency of recombination in this fungus in nature is still unknown because the sexual stage of *Leptographium longiclavatum* has never been observed and the population structure was found to be largely clonal using multiloci sequences [32].

In this investigation, we describe the population genetics structure of *L. longiclavatum* in North America and compare it to *G. clavigera* and their common vector MPB. Since two and four genetic clusters were revealed from MPB and *G. clavigera*, respectively [8], [38], we used population genetics approaches to address the following questions: (1) Are populations of *L. longiclavatum* geographically structured and recombining in western Canada? (2) Do populations conform to isolation-by-distance patterns, and if so, at what scale? (3) Do the fungal symbionts and the MPB have congruent population structures? By analyzing the genetic structure of *L. longiclavatum* and comparing it with *G. clavigera* and their beetle vectors, we can infer the ecological and evolutionary processes shaping their population structures such as historical isolation and drift, offering insights into demographic expansion patterns and improving management strategies for beetle epidemics.

## Materials and Methods

### Ethics statement

Most of our sampling was conducted on crown land, where permits are not required for sampling trees and tree pests for scientific purposes. Permits were also obtained when required to cover both the collection and transport of fungal materials. Parks Canada provided permits for the mountain parks Yoho. Alberta Tourism, Parks, and Recreation provided permits for collection in the Kakwa, Cypress Hills and Canmore. We also received permission from Tembec to collect in their forests around Sparwood. We sampled from dead or dying trees in the middle of the largest forest pest outbreak in Canada's history and no specific permission were required for these activities. We did not work on endangered species.

### Fungal isolates

We sampled a total of 241 fungal isolates from BC and AB, Canada. Fungi were isolated directly from MPB mycangia (specialized membranous invagination on beetle head)/exoskeleton and beetle galleries within the infected lodgepole pines from 17 locations (Figure 1, Table 1, S1) between 2002 and 2009 [34], [39], [40]. The 17 sampling locations (Table 1) consisted of 6 populations previously assigned to *G. clavigera* cluster “North” (TUR, GRP, FCR, FAR, KAK, VAL in northern BC and AB), 5 from the cluster “Rocky” (GOL, YOH, CAN, SPA, CRP in the Rocky Mountains), and 1 from cluster “SBC” (MP in southern BC) [8]. The locations of Burns Lake and Prince George are also geographically close to Houston and Fort St. James in the previously characterized *G. clavigera* populations [8], thus allowing direct comparison between the population structures and genetic clusters inferred from these two fungi.

**Table 1:** Summary information of 17 populations of *L. longiclavatum*, including sampling locations and time, host origin, N (sample size), He (genetic diversity), allelic diversity, % polymorphic loci, and I_A_ (index of association).

Location	Code*	Hosts	Year of isolation	Number of isolates	Number of isolates (clone corrected)	Genetic diversity (He)	Allellic diversity	Percentage of polymorphic loci	I_A_ (index of association)
**Burns Lake, BC**	BL	*Pinus contorta*	2004	9	8	0.424 (0.075)	2.4 (0.34)	90%	−0.3 (0.85)
**Quesnel, BC**	QUE	*Pinus contorta*	2004	12	11	0.401 (0.068)	2.6 (0.40)	100%	0.4 (0.07)
**Prince George, BC**	PG	*Pinus contorta*	2004	18	17	0.349 (0.086)	2.7 (0.616)	90%	0.17 (0.13)
**Kamloops, BC**	KAM	*Pinus contorta*	2004	26	22	0.368 (0.073)	3.1 (0.526)	100%	0.15 (0.82)
**Merrit, BC**	MER	*Pinus contorta*	2009	15	14	0.465 (0.064)	3 (0.447)	100%	0.26 (0.1)
**Williams Lake, BC**	WL	*Pinus contorta*	2004	22	21	0.409 (0.086)	2.7 (0.396)	80%	0.5 (0.0067**)
**Valemount, BC**	Val	*Pinus contorta*	2007/2008	8	8	0.383 (0.066)	2.1 (0.18)	90%	0.35 (0.11)
**Golden, BC**	GOL	*Pinus contorta*	2004	8	7	0.391 (0.069)	2.2 (0.327)	90%	−0.02 (0.46)
**Yoho, AB**	YOH	*Pinus contorta*	2007/2008	5	5	0.349 (0.019)	1.7 (0.26)	50%	0.07 (0.47)
**Canmore, AB**	CAN	*Pinus contorta*	2007/2008	12	11	0.332 (0.079)	2.5 (0.522)	90%	−0.23 (0.79)
**Sparwood, BC**	SPA	*Pinus contorta*	2007/2008	6	6	0.291 (0.101)	1.8 (0.327)	50%	−0.21 (0.8)
**Crowsnest Pass, AB**	CRP	*Pinus contorta*	2007/2008	5	5	0.391 (0.096)	2.1 (0.314)	70%	−0.27 (0.34)
**Fairview, AB**	FAR	*Pinus contorta/Pinus contorta x P. banksiana hybrid*	2007/2008	15	14	0.276 (0.065)	2.4 (0.427)	90%	0.07 (0.34)
**Fox Creek, AB**	FCR	*Pinus contorta/Pinus contorta x P. banksiana hybrid*	2007/2008	17	15	0.276 (0.065)	1.9 (0.277)	70%	0.33 (0.05*)
**Tumble Ridge, BC**	TUR	*Pinus contorta/Pinus contorta x P. banksiana hybrid*	2007/2008	21	17	0.306 (0.067)	2.3 (0.335)	90%	0.08 (0.26)
**Grande Prairie, AB**	GRP	*Pinus contorta/Pinus contorta x P. banksiana hybrid*	2007/2008	23	20	0.286 (0.073)	2.2 (0.249)	90%	0.33 (0.04*)
**Kakwa, AB**	KAK	*Pinus contorta*	2007/2008	19	17	0.336 (0.081)	2.5 (0.543)	80%	0.185 (0.14)
**Total**				241	218	0.323 (0.018)	2.365 (0.096)		0.1 (0.01*)

The null hypothesis of random association of alleles in random mating (I_A_  = 0) was tested by comparing the observed value of the statistic with that obtained after 500 randomization to simulate distribution (* p value <0.05; ** p value <0.01).

*used in manuscript text and figures.

### DNA extraction, PCR, and microsatellite genotyping

DNA was extracted using the methods described in [40], [41]. Microsatellite (simple sequence repeats: SSR) markers developed for *G. clavigera* [42] were used to genotype a panel of eight *L. longiclavatum* isolates (data not shown). Ten polymorphic markers were selected for the current investigation (Table 2).

**Table 2:** Summary of *F_ST_* and heterozygosity (*H*) from each locus in *L. longiclavatum* populations (clone corrected data, *n* = 218).

Locus	Alleles per locus	*F_ST_*	*H*	
			Mean	SE
SR7	13	0.153	0.746	0.026
SR14	4	0.181	0.178	0.045
SR16	2	0.188	0.382	0.033
SR24	4	0.199	0.417	0.055
SR36	3	0.118	0.242	0.044
SR45	6	0.265	0.262	0.064
SR47	4	0.219	0.433	0.032
SR52	3	0.146	0.149	0.040
SR53	6	0.380	0.383	0.059
SR57	3	0.308	0.315	0.036
Overall mean		0.195	0.302	0.017
SE		0.020		

PCR amplification reactions were carried out in either 10 or 14 µL with regard to various markers. Reaction mixture of 10 µL contained 1× PCR buffer, 200 µM each dNTP, 1 pmol of each primer, 0.5 µL labelled M13 (IRDye; LI-COR), 1 µL of AmpliTaq DNA Polymerase (Foster, CA, Invitrogen) and 20 ng of template DNA. Reaction mixture for 14 µl contained 0.71× PCR buffer, 25 mM each dNTP, 0.71 mM MgCL2, 0.71 µM DMSO, 0.71 µM primer, 0.35 µM of each primer (IRDye; LI-COR), 0.35 U of AmpliTaq DNA Polymerase (Foster, CA, Invitrogen), and 5 ng of template DNA. The condition for PCR amplifications was described in [42].

Genotyping was performed on the LI-COR 4300 DNA analyzer on denaturing polyacrylamide gels with molecular size standards 50–350 bp (IRD-700/800 dye) (LI-COR) and analyzed using the LI-COR SAGA software version 2. The holotype of *L. longiclavatum* (SL-KW 1436) has been used as the reference isolate to ensure genotyping consistency.

### Data analysis

#### Population genetic analysis

A matrix of 218 isolates × 10 microsatellite loci was generated. The data was clone-corrected by removing all the repeated genotypes (multilocus haplotypes) from a single geographic population (Table 1). Polymorphisms of each microsatellite locus were assessed by calculating the number of alleles and F_ST_. Heterozygosities, gene diversity, number of alleles and private alleles were estimated across all loci for each population using GenAlEx6 [43].

We analyzed the spatial genetic structure of *L. longiclavatum* by distance-based methods including Principal Coordinates Analysis (PCoA) implemented in GenAlEx6 and AMOVA in Arlequin v.3.11 [44]. Nei's unbiased genetic distance [45], [47] was calculated among all pairs of populations and visualized by PCoA. A Splits network was also generated using Splits Tree4 [46] based on Nei's unbiased genetic distances [45] calculated in Microsatellite Analyzer (MSA) [47].

AMOVA estimates the hierarchical partitioning of genetic variation among populations and regions [43]. The statistical significance of φ-statistics was tested based on 1,000 permutations (default setting). The level of genetic differentiation among *L. longiclavatum* populations was also quantified using F_ST_ [48]. Pairwise F_ST_ was calculated and evaluated using a randomization test with 1,000 iterations using Arlequin v. 3.11 [44].

Investigation of the mating-type (*MAT*) locus evolution and sexual reproduction in *Grosmannia* species demonstrated the presence of two *MAT* alleles in *L. longiclavatum* populations at almost equal frequencies [49]. To test for the hypothesis of random mating, index of association (I_A_) was computed in the populations using Multilocus 1.3b [50]. I_A_ allows the calculation of linkage disequilibrium within populations. When there is no linkage disequilibrium because of frequent recombination event, the value of I_A_ would approach zero [51]. As linkage disequilibrium increases in asexual or inbreeding populations, the value of I_A_ increases [52]. The null hypothesis of random mating was calculated by comparing the observed data with 500 randomized data sets [50].

#### Population structure inference

The population structure and genetic clustering of *L. longiclavatum* were inferred through three complementary approaches: STRUCTURE, TESS, and Bayesian spatial analysis (BAPS). These analytical tools utilize Bayesian clustering algorithms that can help construct population expansion models as well as evolutionary and ecological processes among populations [53]. These analyses were performed concurrently to compare and estimate the robustness of the genetic clusters and population structure.

STRUCTURE 2.3.3 is a model-based clustering method to analyze the association of individual isolates from different geographical locations, to infer clusters and to test for population admixture [54]. The program estimates allele frequencies in each group and population relationships for every individual given the number of clusters (*K*) [55]. It also uses a Monte Carlo Markov Chain (MCMC) to group individuals into *K* distinct populations that minimize Hardy-Weinberg disequilibrium between loci within groups by including prior information, for example, the geographical location of populations [54], [55]. The number of clusters (*K*) was defined from 1 to 12, and each run was conducted with the admixture and correlated allele frequency model, with 100,000 MCMC generations burn-in period followed by 900,000 MCMC generations. Three to five independent runs were performed, and the delta log likelihood value for each *K* was determined to ensure convergence and consistency among runs. The spatial sampling location of each population was included as input *prior*. We used Structure Harvester <http://taylor0.biology.ucla.edu/structureHarvester> to estimate the optimal value of *K* using the *ΔK* method (rate changes in the log likelihood) as in [56].

TESS 2.3 [57] was used to estimate the number of genetic clusters (*K*) present in the data by incorporating geographical coordinates of individuals as prior information to detect discontinuities in allele frequencies. We used an admixture model (CAR) with a burn-in of 10,000 iterations period followed by 60,000 iterations from which estimates were obtained. We performed five independent runs on each *K* (*K* = 2–10) with spatial interaction influence ψ at 0.6 (default value). The optimal value of *K* was determined by the lowest value of the deviance information criterion (DIC). Data from TESS were also visualized graphically using the software DISTRUCT [58] with the optimal value of *K*.

BAPS v. 5.2 uses a stochastic optimization procedure rather than the MCMC algorithms used in STRUCTURE and TESS [59]. We used the “clustering of group” option in BAPS, in which clusters are formed by assembling all the populations with or without the spatial information on each sampling population. The program was run for each number of clusters (*K*) from 2 to 17, with five replicates for each *K*. The output of BAPS was used to perform an admixture analysis to determine individual coefficients of ancestry with regard to the inferred number of clusters. In the admixture analysis, 100 iterations were used to estimate the admixture coefficient for individuals. We also used 100 reference individuals from each cluster, and we repeated the admixture analysis 50 times per each individual.

Isolation-by-distance (IBD) was tested by calculating the correlation between genetic and geographical pair-wise distance matrices in GENEPOP (with ISODE) [60]. Significance of the correlations was tested using 1,000 random permutations by default.

#### Individual assignment and migration pattern

In order to investigate the origin/source of the fungal populations, in particular in newly expanded populations, GENECLASS 2.0 [61] was used to estimate the probability of individual assignment to each sampling population. The standard criterion described by [62] was used to analyze the probability of individuals coming from each area, and we used the method of [63] to simulate 1,000 individuals per regional group of samples. Each individual was assigned to the group that had the highest probability of being the source.

Since MPB was only recently introduced in northern AB and in many parts of northern BC, we used BOTTLENECK v. 1.2 [64] to determine if there was an excess (a recent population bottleneck) or deficit (a recent population expansion) in genetic diversity (H) relative to the number of alleles present in *L. longiclavatum* populations. We analyzed the 17 populations individually and we also pooled the 17 populations into major genetic clusters identified using the distance and Bayesian clustering methods described above. The Sign and Wilcoxon significance tests were used to determine whether loci displayed a significant excess of H > HEQ (genetic diversity expected under mutation drift equilibrium) or deficit in gene diversity under a mutation drift equilibrium for loci evolving under the infinite allele model (IAM), stepwise mutation model (SMM) and two-phase mutation models (TPM) (70% SMM and 30% IAM) [64], [65].

Based on the historical knowledge of the presence of MPB in northern BC and AB, as well as the results obtained with Bayesian clustering methods, we compared the posterior probabilities of three competing scenarios that could have given rise to the epidemic populations of *L. longiclavatum* by approximate Bayesian computation (ABC) [66], [67]. ABC analysis was carried out with DIYABC v.2 [68] (available at http://www1.montpellier.inra.fr/CBGP/diyabc/) using the pooled population data set of major genetic clusters (from 17 sampling populations) inferred from the Bayesian clustering methods and distance methods described above.

We considered three competing scenarios that differed in the order of population divergence, as well as the origin of admixed individuals in epidemic populations (Figure S1). The ABC analysis was performed using parameter values drawn from prior distributions described and by simulating 1.5 million microsatellite data sets in each competing scenario (Table S2). For SSR loci, we followed a generalized stepwise model (GSM) in which a mutation increases or decreases the number of repeated motifs by one or several units, with this number of units being drawn from a geometric distribution [68]. The ABC method is based on summary statistics calculated from the data to represent the maximum amount of information in the simplest possible form. For each population and each population-pair, we estimated the mean number of alleles per locus and the mean expected heterozygosity. The other statistics used were the pairwise F_ST_ values and maximum likelihood estimate of admixture proportion. Then the posterior probability of each scenario was estimated using logistic regression on the 0.1% of the simulated data set closest to the observed data set.

We compared the scenarios by calculating their relative posterior probabilities by polychotomous logistic regression [68] from the 0.1% of simulated data sets most closely resembling the observed data set (in terms of the calculated Euclidean distance between the target and observed summary statistics). The posterior distributions of parameters were then estimated under the most likely scenario by the logit transformation of parameters and linear regression on the 0.5% of simulated data sets most closely resembling the observed data set. The Type-I error was estimated as the proportion of instances in which the selected scenario did not give the highest posterior probability among the competing scenarios, for 500 simulated data sets generated under the best-supported model [69]. We also estimated the Type-II error by calculating the mean proportion of instances that the best-supported model was selected as the most likely model based on the 500 simulated data sets [69].

#### Concordance of population structure patterns among *L. longicavatum, G. clavigera* and MPB

To identify the congruent patterns among the population genetic structures of *L. longicavatum, G. clavigera* and mountain pine beetles (MPB), we compared the significance of congruence among genetic distance matrices with CADM (congruence among distance matrices) [70] using the ape package version 3.0–11 [71] in the R framework. The test applied in CADM is considered an extension of the Mantel test and allows the comparison of more than two distance matrices [70]. The geographic distance among sampling locations was also included to identify potential spatial autocorrelation. Owing to the variation in the sampling locations among the two fungal symbionts and MPB, a subset of 14 populations was selected (Quesnel, Kamloops and Merritt were excluded) to compute the genetic matrices among *L. longiclavatum*, *G. clavigera* [8] and MPB [38].

## Results

### Genetic variation and linkage disequilibrium

Genetic diversity (H_e_) for all the samples and each population separately was high, with values ranging from 0.276 (Fox Creek/Fairview, AB) to 0.465 (Merritt, BC), and overall allelic diversity ranging from 1.7 (Yoho, BC) to 3.1 (Kamloops, BC) (Table 1). In general, populations in British Columbia had greater gene diversity (0.291–0.465) than those from the Rocky Mountains in AB (0.291–0.391), or northern AB and BC (0.276–0.336) (Table 1). The number of privates alleles was also higher in populations in British Columbia (11) than in the Rocky Mountains (5) and northern BC and AB (0). Among the 218 studied isolates (Table S1), we identified 176 (i.e., 80%) unique multilocus haplotypes, with clonal multilocus haplotypes (i.e., identical multilocus haplotypes within the same location) occurring infrequently.

The number of alleles per locus varied between 2 and 13, and gene diversity per locus ranged from 0.149 to 0.746 (Table 2). The proportion of total genetic diversity attributed to population differentiation ranged from 0.118 to 0.380 for the 10 loci, with an overall average of 0.195 (Table 2).

Only 3 out of 17 populations had an index of association (I_A_) that deviated significantly from 0 (Table 1), suggesting that recombination is occurring in 15 populations. However, the I_A_ of the total sample deviated significantly from 0, suggesting population differentiation that may be the result of Wahlund effect [52].

### Population structure and differentiation

In general, the various Bayesian clustering algorithms showed similar population structure patterns with at least three genetic clusters, regardless of the weightings of spatial location *priors* (Figure 1, S2, S3). STRUCTURE analysis inferred the existence of three clusters based on the Evanno method implemented in Structure Harvester (Figure 1, S2). The level of individual assignment and admixture was also explored and examined for *K* = 2 and *K* = 4 to ensure the optimal cluster *K* capture most of the biological/genetic characteristics of the population structure. Similar to the genetic clusters detected in *Grosmannia clavigera* [8], we named the three clusters British Columbia (“BC”), containing populations of BL, PG, QUE, WL, KAM, and MER; “North”, comprising populations of TUR, FCR, GRP, FAR, and KAK in conifer stands where MPB has been only established in the past decade, and the remaining populations “Rocky” (Figure 1). The “North” cluster was more homogeneous; 90% of the individuals from all populations have very high membership coefficients to one cluster (>0.900; Figure 1, Table S3). The “Rocky” cluster (90% of individuals) also has relatively high membership coefficients (>0.8). In contrast, the “BC” cluster was more heterogeneous, with a greater level of admixture. Only about 37% of individuals from “BC” had a membership coefficient of >0.9 (Table S3).

BAPS produced an optimal partition of six population groups with or without the prior spatial information of sampling populations (Figure S2). The “North” cluster was recovered as the one in STRUCTURE. However the “BC” cluster detected by STRUCTURE was divided into three groups, while the remaining two clusters corresponded to the “Rocky” cluster in STRUCTURE, divided into a northern and a southern group (Figure S2). In contrast to results of STRUCTURE and TESS, the Valemount population belonged to the “North” cluster instead of the “Rocky” one. Using the admixture analysis implemented in BAPS, a lower level of admixture was found in each cluster compared with those in STRUCTURE.

The software TESS, which accounts for spatial patterns in genetic structure, also generated similar population clustering patterns. The TESS runs with the smallest DIC values showed that the number of clusters (*Kmax*) was greater than 3 (Figure S3). Populations in the “North” formed a unique cluster with a lower level of admixture, while the remaining populations belonged to two genetic clusters showing admixed ancestry in many populations and isolates. The model-based clustering results in TESS and adjusting spatial interaction factor values (ranging from 0. 1 to 0.8) had no influence on the optimal number of cluster *K* estimated in the analyses.

**Figure 2:**
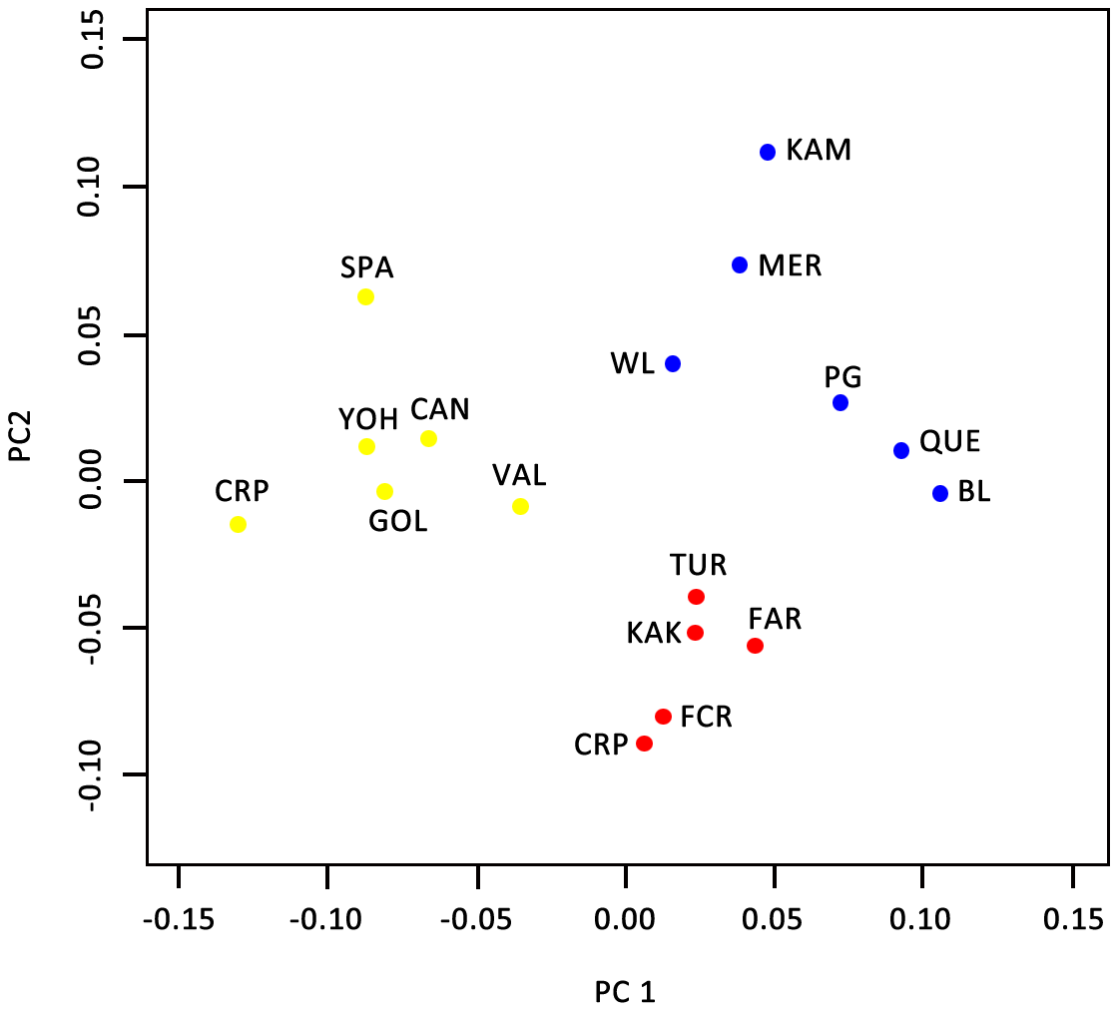
Principal Coordinates Analysis (PCoA) among 17 *Leptographium longiclavatum* populations based on Nei's genetic distance using GenAlEx (cluster “BC” by blue, “North” by red, “Rocky” by yellow). The first and second accounted for 37% and 22.2% of the variance, respectively.

The PCoA produced a distribution pattern similar to the Bayesian inference and suggested the presence of three clusters with the two hypothetical axes explains 37.7 and 22.2% of the total variation respectively (Figure 2). The geographic populations within these three clusters were almost identical. A Splits network indicated a strong differentiation among populations with the presence of recombination (Figure S4). Populations in BC and Rocky Mountain had relatively long branches, while those in the “North” were rather close to each other.

The analysis of molecular variance (AMOVA) performed on the 17 populations indicated that 13.42 and 86.58% of the genetic variation was attributed to variations among and within populations, respectively (*p*<0.001) (Table 3). To further investigate differentiation among the three genetic clusters established on the basis of the Bayesian and distance analyses, AMOVA attributed 8.39, 7.32 and 84.28% of the total variation to variations among clusters, among locations within clusters and among individual isolates within populations, respectively, all of which were highly significant (*p*<0.001) (Table 3).

**Table 3:** Analysis of Molecular Variance (AMOVA) for *Leptographium longiclavatum* populations based on (i) sampling locations and (ii) three genetic clusters pooled according to the PCoA and STRUCTURE (Degrees of freedom (df), sum of squares (SS), variance estimates, percentages of total variation (%) contributed by populations, clusters and individual isolates within populations are shown).

(i)	df	SS	Variance	%	*p*-values
Among all 17 populations	16	157.325	0.309	13.42	<0.001
Within each of 17 populations	419	835.895	1.995	86.58	<0.001
Total	435	993.22	2.304	100	
(ii)					
Among 3 Clusters	2	68.242	0.1987	8.39	<0.001
Among populations within 3 clusters	14	89.082	0.1734	7.32	<0.001
Within 17 populations	419	835.895	1.995	84.28	<0.001
Total	435	993.22	2.3670	100	

**Figure 3:**
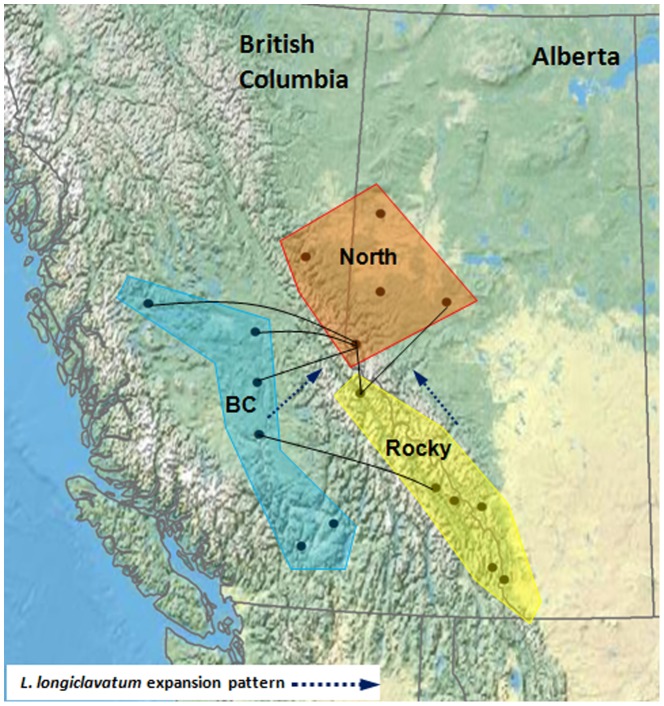
Diagram showing potential gene flow among three clusters and the movement and demographic expansion pattern of *L. longiclavatum* inferred from DIYABC. Solid lines indicate there is no significant difference between the F_ST_ values of two locations, suggesting gene flow between them (only connections among clusters are shown; refer to Table S4 for F_ST_ differentiation/connections among locations within each cluster).

Significant population pair-wise F_ST_ was common across geographic groupings, suggesting strong genetic differentiation (Table S4, Figure 3). With the exception of populations “Kamloops” and “Sparwood” within the corresponding “BC” and “Rocky” clusters, pair-wise genetic differentiations (F_ST_) between populations generally were non-significant within the three geographic groupings, supporting their genetic similarities. Fairview and Yoho (F_ST_ = 0.3271, *p*<0.001) and Kakwa and Fox Creek (F_ST_ = −0.0005, *p* = 0.9) were the most and least differentiated populations, respectively (Table S4) indicating the presence of isolation-by-distance in the entire data set. However, Kakwa and Valemount were not differentiated significantly from 8 and 6 of the 17 populations, respectively (Table S4, Figure 3).

Low genetic distance estimates were observed among populations within the “North” cluster. However, populations collected from the recent epidemic area and within the “Rocky” cluster were genetically less differentiated (Figure S4). These results indicate that geographic distance affects the population structure. Significant correlation between genetic (F_ST_/1-F_ST_) and geographic (Km) distances was observed for the entire data set (r^2^ = 0.184, *p*<0.001), as well as the data set containing all populations in BC and the Rocky (r^2^ = 0.36, *p*<0.001); however, there was no strong evidence of isolation by distance within each of three genetic clusters; ‘BC’ (r^2^ = 0.11, *p* = 0.124), “Rocky” (r^2^ = 0.073, *p* = 0.114) and “North” (r^2^ = −0.069, *p* = 0.111) (Figure S5).

### Individual assignment, migration and demographic history

Results from GENECLASS produced an overall low correct assignment rate to home (24%) (i.e., the 17 original sampling locations). Populations belonged to one of three assignment classes: 1) generally high (3 populations equal to or above 50 to 80%), 2) low (8 populations ranged from 45.5 to 12.5%), and 3) extremely low (6 populations below 6% to zero) (Table 4).

**Table 4:** Results of individual assignment from GENECLASS.

Location	# Sample	# Assigned home^a^	% Home^b^	% Cluster^c^	Ratio^d^
**Burns Lake**	8	4	50.00	100.00	**5.8**
**Quesnel**	11	4	36.36	100.00	1.48
**Prince George**	17	1	5.88	100.00	1
**Kamloops**	22	10	45.45	95.45	**2.45**
**Merritt**	14	9	64.29	100.00	**5.18**
**Williams Lake**	21	8	38.10	100.00	**2.12**
**Valemount**	8	1	12.50	12.50	0.13
**Golden**	7	0	0.00	14.29	0
**Yoho**	5	4	80.00	80.00	1.4
**Canmore**	11	4	36.36	45.45	0.5
**Sparwood**	6	2	33.33	33.33	0.1
**Crowsnest Pass**	5	2	40.00	40.00	0.09
**Fairview**	14	3	21.43	28.57	0.64
**Fox Creek**	15	0	0.00	26.67	0
**Tumbler Ridge**	17	0	0.00	23.53	0
**Grande Prairies**	20	0	0.00	5.00	0
**Kakwa**	17	0	0.00	11.76	1

a Number of individuals assigned to original sampling location (correct assignment).

b Assignment rate to original location ( =  Nhome /N).

c Assignment rate to the genetic cluster recognized from STRUCTURE.

d Ratio  =  total number of individuals (out of 218) assigned to the location / sample size of the location (N).

The overall rate of individual assignment to each of the three inferred clusters increased substantially to a minimum assignment of 95% in “BC”, but remained fairly low in “North” ranging from 5 to 28% in 5 populations, indicating a low level of differentiation among populations within the same genetic cluster (Table 4). Out of the 17 sampling locations, Burns Lake and Merritt have very high assignment ratios (5.8 and 5.18, respectively), followed by Kamloops (2.45) and Williams Lake (2.12), all located in ‘BC’. In contrast, 9 of 11 remaining populations from “North” and “Rocky” received a very low assignment (<1 or even 0).

The possibility of population bottlenecks/founder events, or population expansion was tested on the 17 populations with the program BOTTLENECK. The Sign and Wilcoxon's tests were used to detect significant excess or deficits in gene diversity [65]. Most samples (15 out of 17 under the IAM and 14 out of 17 under the TPM) had more loci showing an expected heterozygosity excess than an expected deficit. Within these samples, nine populations and five populations deviated significantly under IAM and TPM, respectively, indicating a population bottleneck. Fairview was the only sample that exhibited a significant expected heterozygosity deficit, indicating a recent population expansion (Table 5). These analyses appeared to be consistent with the history of MPB population expansion. The general lack of significance may be caused by the lack of power specifically when fewer than 20 individuals are used [65].

**Table 5:** Comparison of observed gene diversity (H) with expected gene diversity (HEQ) at mutation-drift equilibrium calculated from the observed number of alleles under the IAM, SMM and TPM for the 17 populations.

		D/E					
	IAM	TPM	SMM		p		mode
**“BC”**	3/7	4/6	8/2	0.38	1	0.08	normal
**Burns Lake**	2/7**	2/7**	2/7	0.004	0.004	0.25	shifted
**Quesnel**	4/5	4/5	4/5	0.3	0.57	0.82	normal
**Prince George**	4/6	4/6	4/6	0.3	0.6	0.9	shifted
**Kamloops**	5/5	5/5	7/3	0.7	0.8	0.2	normal
**Merritt**	1/9**	2/8	4/6	0.01	0.1	0.375	normal
**Williams Lake**	1/7*	2/6	3/5	0.02	0.07	0.46	normal
**“Rocky”**	4/6	5/5	6/4	0.23	0.4	0.2	normal
**Golden**	3/6*	3/6*	3/6*	0.02	0.02	0.02	shifted
**Sparwood**	0/5*	0/5*	0/5*	0.03	0.03	0.03	shifted
**Yoho**	0/5*	1/4	1/4	0.03	0.06	0.2	shifted
**Canmore**	4/5	4/5	4/5	0.49	0.6	1	normal
**Crowsnest Pass**	0/7**	2/5*	2/5	0.008	0.03	0.07	shifted
**Valmount**	3/6*	3/6*	3/6	0.03	0.04	0.4	shifted
**“North”**	4/6	5/5	5/5	0.37	1	0.3	normal
**Tumbler Ridge**	3/6	3/6	3/6	0.4	1	1	normal
**Fairview**	4/5	8/1*	8/1**	1	0.02	0.01	normal
**Fox Creek**	¾	3/4	3/4	0.11	0.4	0.2	shifted
**Grande Prairies**	5/4	5/4	6/3	0.6	1	0.9	normal
**Kakwa**	1/7*	1/7	2/6	0.04	0.07	0.6	normal

IAM, infinite allele model; SMM, stepwise mutation model;

TPM, two-phase mutation models.

† D / E is the number of loci showing a deficit / excess of gene diversity. Significance estimates of excess or deficiency across loci were obtained using the one-tailed Wilcoxon test and the sign test.

*P≤0.05, **P≤0.01.

The recent demographic expansion of MPB populations may have left a signature on the genetic structure of its fungal associate, *L. longiclavatum*. We used the ABC method to compare the alternative evolutionary scenarios of the demographic histories with our data. The DIYABC analysis provided the strongest support for scenario 3 (Probability  =  0.45; Table 6) but scenarios 1 and 2 which assumed no admixtures also received some support. Scenario 3 assumed that the *L. longiclavatum* population diverged early between ‘Rocky’ and ‘BC’ (Figure S1, Table 6, S2), and that the epidemic population ‘North’ originated from ‘Rocky’ with admixed individuals from ‘BC’. Despite the higher posterior probability of scenario 3 as compared with the other two scenarios, 55.6% of the 500 pseudo-observed data sets simulated with scenario 3 were incorrectly identified as having been generated under scenario 1 (29.4%) or 2 (26.2%) (Type-I error) (Table 6). Of the 500 pseudo-observed data simulated using each of the two alternative scenarios (1 and 2), 17.8 and 21% were incorrectly identified as having been generated under scenario 3 (38.8% Type-II error rate) (Table 6).

**Table 6:** Model choice and robustness on model choice for discrimination among three scenarios (SC).

	SC1 (BC > Rocky > Epidemic)	SC2 (Rocky > BC > Epidemic)	SC3 (BC + Rocky > Epidemic)
Posterior probability	0.2077 [0.2015–0.2140]	0.3652 [0.3574–0.3731]	0.4270 [0.4195–0.4345]
**Performance evaluation (N = 500)**			
**D (SC1)**	0.78	0.052	0.294**
**D (SC2)**	0.042	0.738	0.262**
**D (SC3)**	0.178*	0.21*	0.444

*Type I error, **Type II error.

### Congruence between fungal symbionts and beetles

We used CADM to test for congruence among the MPB genetic distance matrix, two fungal genetic distance matrices and a geographic distance matrix (Table 7). The global CADM test rejected the null model of incongruence among all the matrices, and the posterior results indicate that all matrices were congruent to each other. Mantel tests demonstrated a highly significant correlation between the genetic distance matrix of *L. longiclavatum* to those of *G. clavigera* and *D. ponderosae* (r = 0.67 and 0.78, *p*<0.001, respectively; Table 7), as well as the matrices between *G. clavigera* and *D. ponderosae* (r = 0.72, *p*<0.001, Table 7). The congruence indicated similar landscape-level population structures. The fungal genetic distance matrices and the MPB genetic distance were also congruent to the geographic distance, suggesting significant spatial correlation (Table 7).

**Table 7:** Congruence among genetic distance matrices of two fungi, the mountain pine beetles and geographic distance among sampling locations using CADM implemented in the R framework.

Global congruence	*Ho*: matrices are incongruent			
**Kendall's W**	0.7975			
**Friedman's χ^2^**	287.1177***			
**A posteriori pairwise congruence**	Ho: matrix is incongruent with remaining matrices			
	H1: matrix is congruent with at least one other matrix			
	MPB	*G. clavigera*	*L. longiclavatum*	Geographic distance
**Mantel.mean**	0.7368	0.7473	0.7040	0.7322
**P-value**	***	***	***	***
**One-tailed Mantel test**	MPB	*G. clavigera*	*L. longiclavatum*	Geographic distance
**MPB**	1	0.7248***	0.6749***	0.8107***
***G. clavigera***		1	0.7840***	0.7330***
***L. longiclavatum***			1	0.6528***
**Geographic distance**				1

## Discussion

### Populations of *Leptographium longiclavatum* are geographically structured and congruent with MPB and *Grosmannia clavigera*

*Leptographium longiclavatum* exhibited a strong population structure in western Canada that corresponds to geographic location [8]. A very similar structure was also identified in the mountain pine beetle and in *G. clavigera*, another fungal symbiont [8], [38], [32], [72], [73]. The symbiotic relationship among *L. longiclavatum, G. clavigera* and MPB may play an important role in shaping their congruent population structure. The highly significant correlation among genetic distance matrices of this vector-symbiont complex highlights the concordance of demographic processes in these interacting organisms sharing a highly specialized niche and supports the hypothesis of long-term multipartite beetle-fungus co-evolutionary history and mutualistic relationships. It is possible that similar patterns exist in other insect-fungal symbiont association. For example, a parallel distribution pattern in insect-symbiont complexes has been reported for another bark beetle-fungus association (*Ceratocystis polonica* (Siemaszko) C. Moreau and *Ips typographus* L.) [74].

The observed population structure (e.g. divergence between BC and Rocky populations) may have several possible explanations. Post-glacial expansion of the MPB followed by population differentiation along re-colonization path has been proposed [72], [75]. Landscape features are likely important contributors in shaping the species associated with the MPB complex. The Rocky Mountains represent a physical and climatic barrier in shaping the geographic distribution by limiting gene flow [38]. Previous studies of the MPB population structure have reported that geographic barriers, including mountain ranges and large distances, play a role in limiting gene flow and causing divergence among populations [38], [72], [75]. The distinct populations in southern BC could be explained by the presence of mountain ranges (west of the Rocky Mountain Trench) that have blocked gene flow as in the southern MPB populations [38].

Given the dispersive nature of MPB and the distance they can travel, the potential for isolated/differentiated vector-symbiont populations should be low [32], even at rather long distance. However, major MPB eruptions have been recorded more than three times in the past century in BC [18]. If remnant populations of fungi and MPB persisted in small regional populations at endemic levels, sufficient time may have passed for population differentiation to occur through genetic drift and bottleneck. The historical genetic signatures of regional differentiation were revealed in BC and Rocky populations when these disjointed populations expanded in the outbreak [32]. Some of these *L. longiclavatum* populations were possibly established following glacial retreat and remained isolated until the current MPB outbreak.

The “North” cluster represents recently established populations at the margin or beyond the historic MPB range. The relatively weak genetic differentiation among *L. longiclavatum* populations could be attributed to the frequent short-distance and long-distance aerial dispersal (at altitudes above the tree canopy), as well as the ‘rainout’ mechanism of MPB invasion and infestation in the past decade [76]. During a ‘rainout’ period in the current outbreak, MPB were carried by strong, warm winds from central-south BC across the geoclimatic barrier of the Rocky Mountains into northern AB, Canada. This long distance dispersal could result in the mixing of various fungal populations from various sources and in the homogenization of the genotypic frequency (reduced differentiation) in various *L. longiclavatum* populations.

The substructured populations of *L. longiclavatum* in northern and southern BC have retained high genetic variability. This is consistent with the multiple MPB epicentres hypothesis and the long-term persistence of beetle populations across BC [38], [77]. Tweedsmuir provincial park (located in west-central BC and south of Burns Lake, BC) has been suggested as the primary epicentre of the current MPB outbreak [77]. The genetic diversity of *L. longiclavatum* in northern and central BC is comparable. If colonization/expansion had only originated from a southern population following the end of the glacial period, we would have observed a decreasing diversity from south to north with increasing latitude [78] as observed in *G. clavigera* and MPB [8], [38], [72]. This contrasting diversity pattern might suggest a centre of diversity for *L. longiclavatum* in central BC or the presence of glacial refugia [79], where the historical gene pool was maintained during the ice age. Further characterization of populations in the USA may help to resolve the genetic variation in population structure patterns and the influence of environmental heterogeneity.

### Gene flow and admixture among *Leptographium longiclavatum* populations

Despite the genetic differentiation among three clusters, the populations were not completely isolated. Gene flow and admixed populations were observed. The individual assignments revealed that individuals in the newly established populations in the “North” cluster (northern BC and Alberta) most probably originated from the “BC” cluster (west of the Rocky Mountains). Gene flow estimations among *L. longiclavatum* populations were consistent with MPB expansion from BC into Alberta through the Pine Pass and Yellowhead Pass (50 km east of Valemount) [80]. The MPB attack discovered in the Peace River region (60 km east of Fairview, AB) came from areas of central BC across the Rocky Mountains [15].

The most probable scenario from our ABC analyses also supports that the newly established population in northern Alberta had been from admixture of populations from BC and the Rocky Mountains. Previous analysis of *G. clavigera* and MPB also revealed a likely western source for the newly established eastern populations [8], [38]. The reduced genetic diversity in the “North” and “Rocky” *L. longiclavatum* populations suggested that the populations introduced into northern BC and Alberta have gone through drift, the founder effect and local adaptation despite multiple, constant MPB introductions.

In this study, the population from Valemount, which is situated on the western slopes of the Rocky Mountains (Figure 3), is genetically heterogeneous because it is ‘grouped’ inconsistently, possibly due to multiple MPB invasions and expansion. A similar pattern was also observed for *G. clavigera* [8]; Valemount was suggested to represent a corridor that facilitated expansion from BC eastward into northern Alberta. The current study also demonstrated a low level of genetic differentiation between *L. longiclavatum* populations from Valemount and the locations along the Rocky Mountains. More importantly, Kakwa a population sampled slightly north of Valemount, displays a ‘star’ pattern, with high genetic relatedness to eight other populations and could play a similar role. The low barriers to migration existing along the Rocky Mountain Trench may serve as sources for the continued spread of beetles carrying *L. longiclavatum* to healthy trees north and east of the Rockies due to its proximity to the mountain passes [8], [80].

### *Leptographium longiclavatum* can reproduce sexually

Fungi can be sexual, asexual or exhibit a mixture of both types of reproduction. The type of reproduction and mating system has been known to affect fungal population characteristics [81]. We observed recombinant genotypes, indicating sexual recombination in *L. longiclavatum* is the source of genetic variation [34] but this may happen in cryptic. The sexual stage of *L. longiclavatum* has never been observed and it has been presumed to reproduce mainly via asexual spores called conidia, while the sexual cycle of *G. clavigera* has been observed sporadically and may be more prevalent [32], [34]. In support of sexual recombination, the two mating types alleles were found at a 1∶1 ratio in the *L. longiclavatum* population [49]. Recombination can generate highly adaptive genotypes that can increase in frequency through clonal expansion [82] and contribute to the success of invasive pests and pathogens [83]. However,

### Implications for multipartite symbiosis and pest monitoring

Concordant signals in the demographic expansion and transmission pattern between *L. longiclavatum* and *G. clavigera* also inferred long-term evolutionary history and co-existence of MPB with these fungal associates as different MPB populations can carry different symbiont fungal species. Pathogen dissemination has been influenced by the range expansions of the vector in many other studies [9], [84]. The expansion pattern of the MPB complex was strongly linked to the rapid northeastern expansion of the beetles in the current outbreak; the high correlation in population structure amongst the beetle and two of its fungal associates reveals an intimated relationship between these organisms. Our findings could confirm/illustrate two important points about bark beetle-fungal symbiotic interactions: (1) the association between fungal symbionts and their vector MPB is very specialized, not incidental or opportunistic; and these fungal symbionts could be transmitted vertically, otherwise diffuse patterns would be observed as in the horizontally transmitted pathogens [85], for instance, these fungi have evolved specific adaptations, such as sticky spores that can be transported by the MPB partner; as a compromise, their dispersal may be constrained by their association with the beetles, and (2) this specialization has evolved and arisen in more than one fungal species that have shared similar ecological niches and close evolutionary relationships.

The correlated population structures among the fungal and MPB symbionts may reflect local adaptation and co-evolutionary processes [14], [86], when considering that a plant pathogen can directly or indirectly affect the vector's fitness during the host infections [87]. Also changes in the vector's pattern may influence the population structure of the transmitted pathogens. The demographic and evolutionary processes underlying invasion have been the subject of considerable attention in recent years [88]. Understanding these processes can help in the development of effective management strategies aimed at preventing biological invasions.

The expansion of the MPB's range to higher latitudes and elevations is further than ever observed before [89]. It will be important to further characterize the genotypes of *L. longiclavatum* populations in the latest regions affected by epidemics and any that can be isolated from the infected jack pines [19]. Given the symbiotic relationships and ecological role of *L. longiclavatum* and *G. clavigera* with the MPB might be different, future research should also investigate and characterize the abilities of these fungi under different environmental conditions including temperature and elevations to determine if they have various adaptations, e.g. different cold tolerance [37].

## Supporting Information

Figure S1 **Graphical representation of three scenarios modelled in DIYABC for demographic expansion patterns of three groups of population (clustering the independent geographic locations) with the posterior probabilities.** Scenario 1 indicated the divergence of “BC” population from the ancestral “Rocky” population. The “North/Epidemic” population emerged from the “BC” population. Scenario 2 is similar to scenario 1, but suggesting the “North” population emerged from the “Rocky” population which diverged from “BC”. In scenario 3, the “North” population was an admixture of “BC” and “Rocky” populations.
(TIF)

Figure S2 **Population structure based on individual assignment inferred from STRUCTURE (** ***K*** **= 2–4) and BAPS (** ***K*** **= 6).** Each individual is represented by a line partitioned into *K* segments that represent the individual's estimated membership fractions in *K* clusters.
(TIF)

Figure S3 **Population structure of** ***Leptographium longiclavatum*** **populations using TESS for** ***K*** **= 4. Each** individual is represented by a thin vertical line, which is partitioned into *K* segments that represent its estimated population group membership fractions. Black lines separate individuals from geographical site locations (labeled as in Table 1).
(TIF)

Figure S4 **Splits network showing relationships of the analyzed populations.** Distances between the populations were estimated by the Nei's distance over 10 microsatellie loc.
(TIF)

Figure S5 **Plot of isolation-by-distance for the entire (all) population, and each of the three genetic “BC”, “North”, and “Rocky” clusters, Y  =  ln (km), X  =  F_ST_/1- F_ST_.**
(TIF)

Table S1 **Microsatellite profiles for 241 strains (strains in grey were not included in the clone corrected data set).**
(XLSX)

Table S2 **Prior distributions and the demographic and historical parameters estimated in DIY ABC analyses.** (N: effective population size; t: divergence time in terms of generation time; r: rate of admixture; μ: mutation rate; P: parameter of geometric distribution; μSNI: mutation rate in single nucleotide instability).
(XLSX)

Table S3 **Admixture analysis for 218 individuals of** ***L. longiclavatum*** **from 17 locations: average membership coefficients in three clusters inferred from STRUCTURE.**
(XLSX)

Table S4 **Pairwise F_ST_ calculated with Arlequin (assessed after 100 permutations).** * *p*<0.05; ** *p*<0.01.
(DOC)
