# Supplementary figures and images for: Population Structure of Mountain Pine Beetle Symbiont Leptographium longiclavatum and the Implication on the Multipartite Beetle-Fungi Relationships
Source: PLoS One. 2014 Aug 25;9(8):e105455. doi: 10.1371/journal.pone.0105455 (PMC4143264; doi:10.1371/journal.pone.0105455)

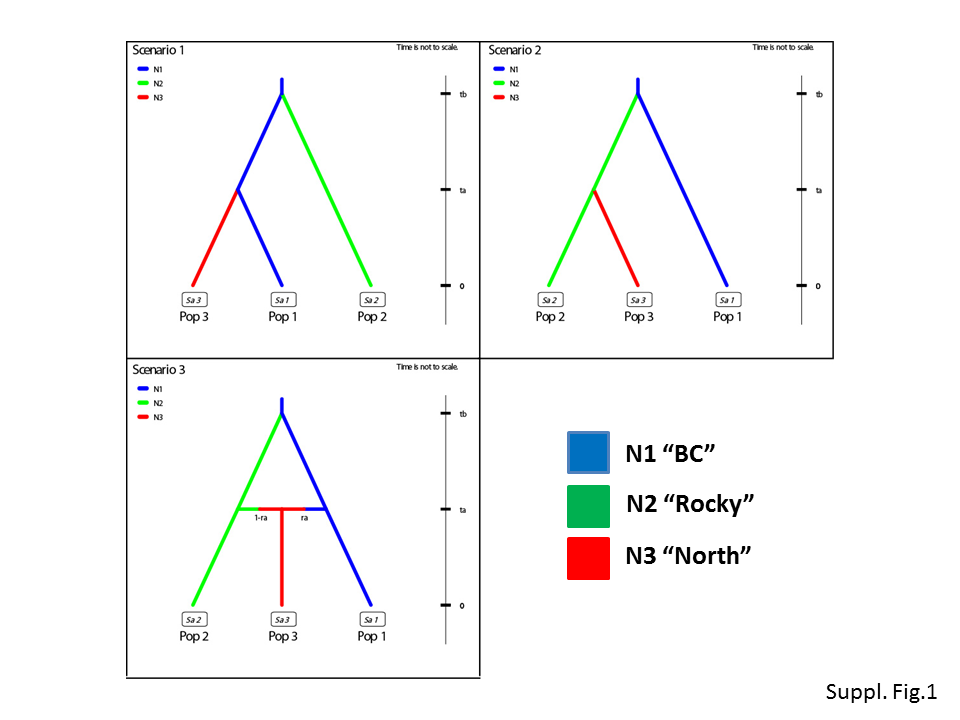

Supplement: Figure S1 — Graphical representation of three scenarios modelled in DIYABC for demographic expansion patterns of three groups of population (clustering the independent geographic locations) with the posterior probabilities. Scenario 1 indicated the divergence of “BC” population from the ancestral “Rocky” population. The “North/Epidemic” population emerged from the “BC” population. Scenario 2 is similar to scenario 1, but suggesting the “North” population emerged from the “Rocky” population which diverged from “BC”. In scenario 3, the “North” population was an admixture of “BC” and “Rocky” populations. (TIF) [file pone.0105455.s001.tif]

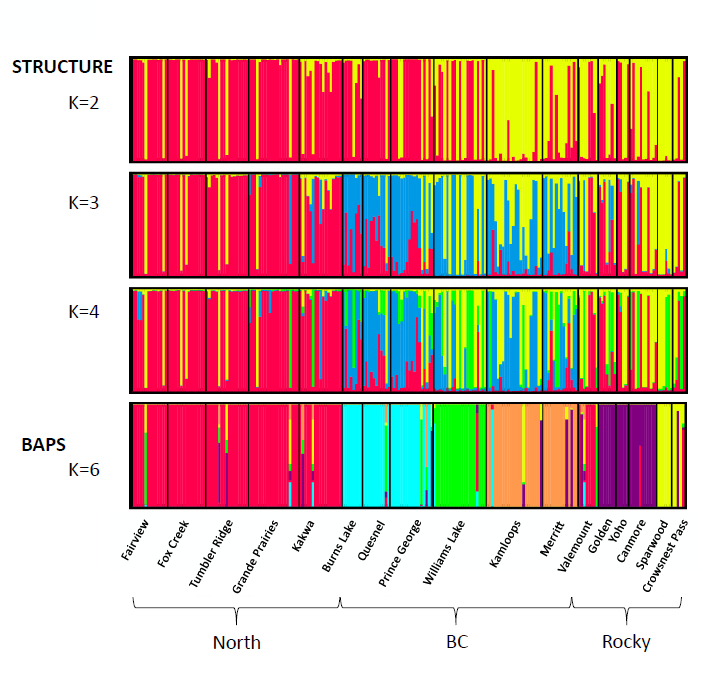

Supplement: Figure S2 — Population structure based on individual assignment inferred from STRUCTURE ( K = 2–4) and BAPS ( K = 6). Each individual is represented by a line partitioned into K segments that represent the individual's estimated membership fractions in K clusters. (TIF) [file pone.0105455.s002.tif]

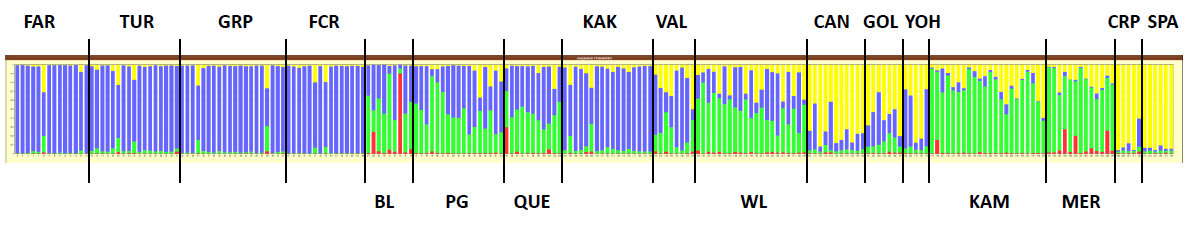

Supplement: Figure S3 — Population structure of Leptographium longiclavatum populations using TESS for K = 4. Each individual is represented by a thin vertical line, which is partitioned into K segments that represent its estimated population group membership fractions. Black lines separate individuals from geographical site locations (labeled as in Table 1). (TIF) [file pone.0105455.s003.tif]

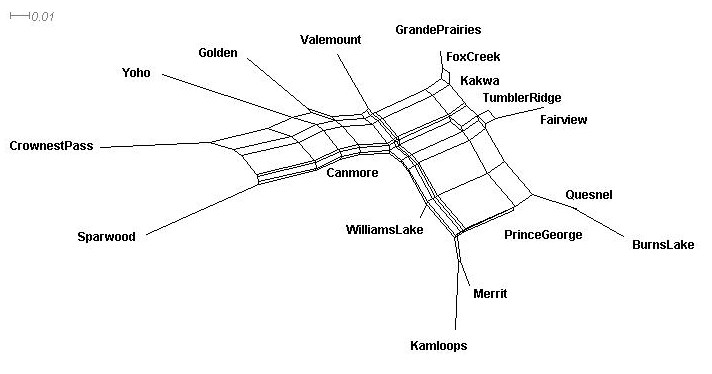

Supplement: Figure S4 — Splits network showing relationships of the analyzed populations. Distances between the populations were estimated by the Nei's distance over 10 microsatellie loc. (TIF) [file pone.0105455.s004.tif]
