# Supplementary material for: Population Structure of Mountain Pine Beetle Symbiont Leptographium longiclavatum and the Implication on the Multipartite Beetle-Fungi Relationships
Source: PLoS One. 2014 Aug 25;9(8):e105455. doi: 10.1371/journal.pone.0105455 (PMC4143264; doi:10.1371/journal.pone.0105455)
Supplement: Table S4 — Pairwise FST calculated with Arlequin (assessed after 100 permutations). * p<0.05; ** p<0.01. (DOC) [file pone.0105455.s009.doc]

Table 4. Pairwise *F_ST_* calculated with Arlequin (assessed after 100 permutations) among *L. longiclavatum* populations. A. 17 Geographic locations. B. 3 Genetic clusters. *F_ST_* values are significant (**p* < 0.05; ** *p* < 0.01). Insignificant *F_ST_*  values are shaded ‘grey’ to indicate potential gene flow among populations.

A.

|  | BL | QUE | PG | KAM | MER | WL | VAL | GOL | YOH | CAN | SPA | CRP | FAR | FCR | TUR | GRP |
| --- | --- | --- | --- | --- | --- | --- | --- | --- | --- | --- | --- | --- | --- | --- | --- | --- |
| Quesnel | -0.003 |  |  |  |  |  |  |  |  |  |  |  |  |  |  |  |
| Prince George | 0.024 | 0.022 |  |  |  |  |  |  |  |  |  |  |  |  |  |  |
| Kamloops | 0.12566* | 0.108** | 0.107** |  |  |  |  |  |  |  |  |  |  |  |  |  |
| Merritt | 0.045 | 0.043 | 0.054 | 0.060 |  |  |  |  |  |  |  |  |  |  |  |  |
| Williams Lake | 0.094 | 0.087* | 0.057 | 0.085** | 0.064 |  |  |  |  |  |  |  |  |  |  |  |
| Valemount | 0.172* | 0.154** | 0.153** | 0.139** | 0.134* | 0.100 |  |  |  |  |  |  |  |  |  |  |
| Golden | 0.196** | 0.189** | 0.197** | 0.175** | 0.112 | 0.122 | 0.089 |  |  |  |  |  |  |  |  |  |
| Yoho | 0.233* | 0.196** | 0.246** | 0.204** | 0.187** | 0.176* | 0.172 | 0.170 |  |  |  |  |  |  |  |  |
| Canmore | 0.177** | 0.162** | 0.137** | 0.131** | 0.106** | 0.115** | 0.122* | 0.079 | 0.146 |  |  |  |  |  |  |  |
| Sparwood | 0.251** | 0.228** | 0.208** | 0.213** | 0.1797** | 0.154* | 0.209* | 0.251** | 0.279* | 0.122 |  |  |  |  |  |  |
| Crowsnest Pass | 0.223** | 0.226** | 0.223** | 0.261** | 0.159* | 0.137* | 0.168 | 0.081 | 0.209 | 0.108 | 0.172 |  |  |  |  |  |
| Fairview | 0.147** | 0.141** | 0.112** | 0.206** | 0.150** | 0.111** | 0.173** | 0.246** | 0.327** | 0.179** | 0.319** | 0.287** |  |  |  |  |
| Fox Creek | 0.130* | 0.121** | 0.124** | 0.237** | 0.177** | 0.147** | 0.124 | 0.173** | 0.241* | 0.142* | 0.286** | 0.226** | 0.107* |  |  |  |
| Tumbler Ridge | 0.127** | 0.114** | 0.107** | 0.168** | 0.130** | 0.115** | 0.132** | 0.171** | 0.213** | 0.106** | 0.260** | 0.239** | 0.016 | 0.065 |  |  |
| Grande Prairies | 0.127** | 0.149** | 0.126** | 0.247** | 0.181** | 0.132** | 0.145** | 0.151** | 0.244** | 0.154** | 0.289** | 0.196* | 0.110** | 0.011 | 0.084* |  |
| Kakwa | 0.076 | 0.076 | 0.072 | 0.162** | 0.121** | 0.106** | 0.087 | 0.140* | 0.198** | 0.096** | 0.224** | 0.214** | 0.061 | -0.001 | 0.031 | 0.033 |

B.

|  | **BC** | **Rocky** |
| --- | --- | --- |
| **Rocky** | 0.05 | -- |
| **North** | 0.06 | 0.12 |
